# T-cell-redirecting therapies in early relapsed multiple myeloma: a systematic review and network meta-analysis

**DOI:** 10.1186/s13045-026-01850-2

**Published:** 2026-10-07

**Authors:** Maximilian Merz, Joao Tadeu Souto Filho, Peter M. Voorhees, Hermann Einsele, Fredrik Schjesvold, Edward R. Scheffer Cliff, Maria-Victoria Mateos, Vania Hungria, Yael C. Cohen, Carlos Fernández de Larrea, Meral Beksac, Ola Landgren, Saad Z. Usmani, Luciano J. Costa, Nico Gagelmann

**Affiliations:** 1 Myeloma Service and Cellular Therapy Service, Department of Medicine, Memorial Sloan Kettering Cancer Center, and Weill Cornell Medical College, 1275 York Ave, New York, NY 10065 USA; 2 Department of Hematology and Cellular Therapy, University Hospital Leipzig, Leipzig, Germany; 3 Faculdade de Medicina de Campos, Rio de Janeiro, Brazil; 4 Levine Cancer Institute, Atrium Health Wake Forest University School of Medicine, Charlotte, NC USA; 5 Department of Internal Medicine II, University Hospital Würzburg, Würzburg, Germany; 6 Department of Hematology, Oslo Myeloma Center, Oslo University Hospital, Oslo, Norway; 7 Department of Clinical Haematology, Peter MacCallum Cancer Centre and Royal Melbourne Hospital, Melbourne, VIC Australia; 8 Sir Peter MacCallum Department of Oncology, University of Melbourne, Melbourne, VIC Australia; 9 Instituto de Investigacion Biomedica de Salamanca, Instituto de Biologia Molecular y Celular del Cancer, Hospital Universitario de Salamanca, Universidad de Salamanca-Consejo Superior de Investigacion, Salamanca, Spain; 10 Clinica São Germano, São Paulo, Brazil; 11 Tel-Aviv Sourasky (Ichilov) Medical Center and Tel Aviv University, Tel Aviv, Israel; 12 Hospital Clínic de Barcelona, Institut d’Investigacions Biomèdiques August Pi i Sunyer (IDIBAPS), Universitat de Barcelona, Barcelona, Spain; 13 Department of Hematology, Ankara Liv Hospital, İstinye University, Ankara, Turkey; 14 Sylvester Comprehensive Cancer Center, Sylvester Myeloma Institute, University of Miami, Miami, FL USA; 15 Division of Hematology and Oncology, Department of Medicine, University of Alabama at Birmingham, Birmingham, AL USA; 16 Department of Medical Oncology, Dana-Farber Cancer Institute, Boston, MA USA; 17 Department of Stem Cell Transplantation, University Medical Center Hamburg- Eppendorf, Hamburg, Germany

**Keywords:** Multiple myeloma, Ciltacabtagene autoleucel, Teclistamab, Talquetamab

## Abstract

**Background:**

Chimeric antigen receptor T cells and bispecific antibodies, are moving from late-line salvage into early relapsed multiple myeloma. Indirect comparisons in heavily pretreated patients have favored cell therapy, but whether that hierarchy holds in early relapse is unknown.

**Methods:**

We analyzed randomized phase 3 trials comparing a T-cell-redirecting therapy against a daratumumab-pomalidomide-dexamethasone-type control after at least one prior line. Pseudo-individual patient time-to-event data were reconstructed from published survival curves. Regimens were compared indirectly in Bayesian network meta-analyses reported under fixed-effect and random-effects assumptions, re-estimated in a frequentist graph-theoretical model. Control-arm dispersion was quantified by random-effects synthesis of median progression-free survival. Overall survival was additionally analyzed as restricted mean survival differences at 24 and 36 months. Safety and a cost-consequence analysis were assessed descriptively (PROSPERO: CRD420261431847).

**Results:**

Four trials (2,174 patients) investigating ciltacabtagene autoleucel, teclistamab-daratumumab, and talquetamab-daratumumab with or without pomalidomide were included. Control-arm median progression-free survival spanned 9.9 to 24.4 months (I-squared 91%). Under fixed effects, teclistamab-daratumumab ranked first for progression-free survival (P-score 98%, the mean probability of outranking a competing treatment rather than the probability of being best) and separated from talquetamab-daratumumab (hazard ratio 0.55, 95% credible interval 0.34 to 0.88). Once between-trial heterogeneity was admitted, every contrast crossed unity (versus ciltacabtagene autoleucel 0.62, 0.21 to 1.81). Overall survival was concordant under both models (teclistamab-daratumumab versus ciltacabtagene autoleucel 0.94, 0.55 to 1.62), with restricted mean survival gains over control of 0.7 to 1.6 months at 24 months and rankings between 56% and 70%. Toxicity profiles differed across modalities, although their overall burden cannot be reliably compared across trials. Cost per progression-free month, computed against a population-standardized denominator, was lowest for cilta-cel and converged with the bispecific regimens only under short, fixed treatment durations.

**Conclusions:**

Randomized evidence in early relapse does not establish an efficacy hierarchy among these regimens. Progression-free survival estimates favored teclistamab-daratumumab, but no contrast was robust to plausible between-trial heterogeneity, and equivalence could not be demonstrated. Until comparative data exist, treatment selection cannot rest on efficacy data alone and will be informed by patient- and disease-related factors, toxicity, sequencing, access, and cost.

**Supplementary Information:**

The online version contains supplementary material available at https://doi.org/10.1186/s13045-026-01850-2.

## Background

T-cell-redirecting therapies have transformed the treatment of advanced multiple myeloma (MM). Chimeric antigen receptor (CAR) T cells directed against B-cell maturation antigen (BCMA), and ciltacabtagene autoleucel in particular, induced deep responses and long-term remissions in patients who have exhausted conventional options [1–4]. Bispecific antibodies targeting BCMA or GPRC5D followed, offering off-the-shelf administration and high response rates [5–9].

In later lines, indirect comparisons and real-world analyses have suggested that CAR T cells may offer greater efficacy despite higher late toxicities [10–12]. These comparisons, however, rest largely on single-arm registrational cohorts or non-randomized data in heavily pretreated populations, and they seldom account for how widely the comparator context varies across studies. Whether such differences would persist in earlier relapse, where each agent has now been tested in a randomized phase 3 trial has not been established. No head-to-head trials exist, and none are likely to be conducted.

We therefore performed a systematic review and anchored network meta-analysis of the randomized early-relapse trials. Rather than ranking the regimens, we aimed to determine whether the randomized evidence supports an efficacy hierarchy and how strongly any apparent difference depends on assumptions about between-trial comparability, complemented by descriptive safety and cost analyses.

## Methods

### Study design

**Fig. 1:**
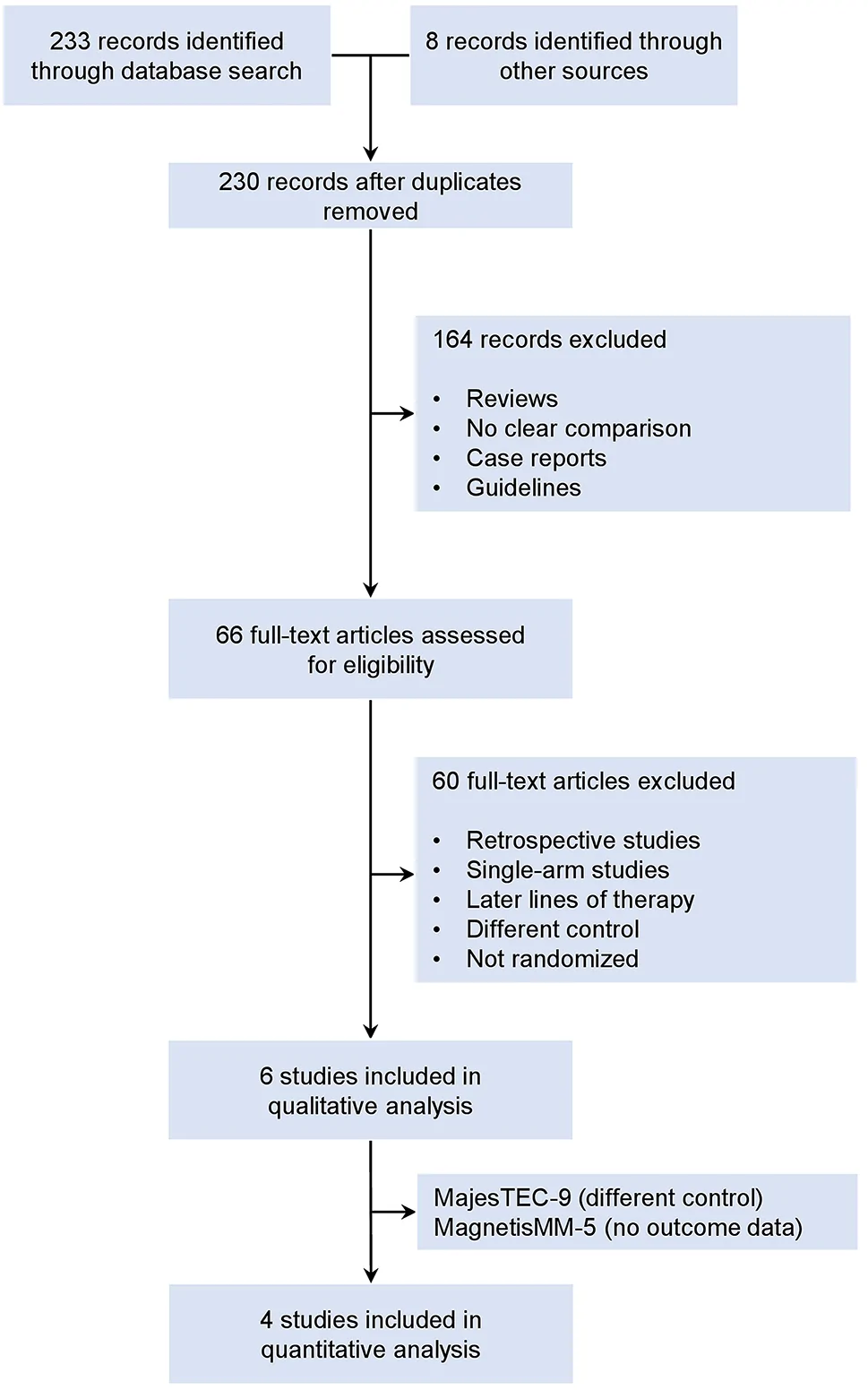
Study selection. PRISMA flow diagram for the systematic review

We conducted a systematic review and Bayesian anchored network meta-analysis comparing BCMA- and GPRC5D-directed T-cell-redirecting therapies in early relapsed/refractory MM (Fig. 1 and Supplemental Methods). Because no head-to-head trials exist, treatments were compared indirectly through their shared daratumumab-pomalidomide-dexamethasone (DPd)-type control arm. The review follows the PRISMA-NMA and EQUATOR guidance [13]. The protocol was registered with PROSPERO (CRD420261431847). Because each experimental regimen was compared with the control in a single trial, the network was star-shaped without closed loops; consistency could not be assessed, and the analysis reduces to anchored indirect comparisons preserving within-trial randomization.

Sources were searched from inception to June 15, 2026, supplemented by ClinicalTrials.gov and annual-meeting proceedings to capture recent phase 3 readouts. Search concepts combined multiple myeloma, relapsed/refractory or lenalidomide-refractory disease, the candidate interventions (bispecific antibodies or CAR-T), a DPd-type comparator, and a randomized-trial filter.

### Eligibility and data extraction

Eligible studies were randomized phase 3 trials in adults with relapsed or refractory MM after one or more prior lines of therapy, with eligibility or enrollment centered on one to three prior lines, evaluating a T-cell-redirecting therapy against a predominantly DPd-based control and reporting an extractable progression-free survival (PFS) or overall survival (OS) hazard ratio or Kaplan-Meier curve. Single-arm trials and trials without a shared DPd-based comparator or extractable time-to-event data were excluded. Idecabtagene vicleucel (KarMMa-3) was therefore excluded, as its investigator’s-choice comparator did not provide a separable DPd anchor.

Three reviewers (NG, MM, JTS) independently extracted trial characteristics, effect estimates, and safety data. Pseudo-individual patient time-to-event data were reconstructed from published Kaplan-Meier curves and numbers-at-risk tables using the Guyot algorithm; no original individual patient data were used. Where cumulative censoring was reported (CARTITUDE-4, APOLLO), reconstruction reproduced reported event totals exactly; for the remaining trials, control-arm curves were reconstructed parametrically from published landmark statistics and numbers at risk, and medians and landmark survival were reproduced to within reading error [14].

### Statistical analysis

The primary endpoints were progression-free survival (PFS) and overall survival (OS). Pre-specified safety endpoints, cytokine release syndrome (CRS), neurotoxicity, and second primary malignancies, were assessed descriptively, as anchored comparison was unlikely. Four analyses were prespecified. First, between-trial heterogeneity of the DPd-type control was quantified by DerSimonian-Laird random-effects synthesis of control-arm median PFS on the log scale (I², τ²) [15]. Second, treatments were compared in a Bayesian anchored network meta-analysis on the log-hazard-ratio scale [16]. Because each edge derived from a single trial, between-trial heterogeneity (τ) could not be estimated and was assigned a weakly informative half-normal prior (standard deviation, 0.32) consistent with empirical distributions for time-to-event comparisons (a τ of 0.32 on the log-HR scale implies that, across trial settings, 95% of true treatment effects would span roughly a 1.9-fold factor in either direction). Fixed-effect (τ = 0) and random-effects estimates were reported side by side to expose the dependence of any separation on the heterogeneity assumption [17]. Third, to confirm that conclusions were not an artifact of the Bayesian framework or its heterogeneity prior, the network was re-estimated in a frequentist framework using the graph-theoretical approach of Rücker with DerSimonian-Laird heterogeneity, and treatments were ranked by P-score, the frequentist analogue of the surface under the cumulative ranking curve. Since the network formed a star with a single study per comparison, no within-network heterogeneity was estimable and the random-effects model reduced to the fixed-effect model, so P-scores index the mean probability that a treatment outranks a competitor rather than a probability of being best [18]. Fourth, OS was analyzed both as an anchored network meta-analysis of hazard ratios and, to avoid the proportional-hazards assumption, as an anchored comparison of restricted mean survival time (RMST) differences at 24 months, extended to 36 months where follow-up permitted [19, 20]. Risk of bias was assessed with Cochrane RoB-2 [21]. The random-effects marginal is a scale mixture of normals over the half-normal prior on tau and was evaluated exactly by quadrature. Overall survival RMST intervals were obtained by bootstrap with 20,000 replicates (fixed seed). 95% credible intervals (CrI) are reported for the Bayesian model and 95% confidence intervals (CI) for the frequentist and RMST analyses., and 95% credible/confidence intervals are reported throughout [22]. The primary half-normal prior (scale 0.32) places the prior median of tau at 0.22 and its 95th percentile at 0.63 on the log-hazard-ratio scale; robustness was examined with half-normal scales of 0.16, 0.50, and 1.00, and by a tipping-point analysis fixing tau across a grid from 0 to 0.50 to identify the smallest value at which a fixed-effect separation no longer excluded 1. To distinguish absence of evidence of a difference from evidence of similarity, we report the posterior probability that each hazard ratio lies within 0.80 to 1.25. MonumenTAL-3 contributed two experimental arms sharing one control arm and was modeled as a three-arm trial. The covariance between its two anchored log hazard ratios was approximated by the inverse of the number of events in the shared DPd arm, and the contrast between the two talquetamab regimens, a within-trial randomized comparison, was estimated without a between-trial heterogeneity term.

### Cost-consequence analysis

We compared drug-acquisition costs relative to progression-free time in a cost-consequence analysis from a US payer perspective, in accordance with CHEERS. This was not a cost-effectiveness or cost-utility analysis, and no quality-adjusted life-years were modeled. Drug-acquisition costs were based on US wholesale acquisition cost for an 80-kg patient without wastage, with cilta-cel modeled as a one-time cost and the bispecific regimens accrued cycle-by-cycle per label dosing at each trial’s observed median treatment duration. The primary denominator was a population-standardized 30-month restricted mean PFS, obtained by applying each regimen’s anchored hazard ratio to a common reference daratumumab-pomalidomide-dexamethasone curve derived from the pooled reconstructed control arms, so that between-trial differences in absolute PFS did not enter the cost ratio. Trial-specific restricted mean PFS served as a secondary denominator. Deterministic sensitivity analyses varied treatment duration, acquisition cost of the CAR T-cell and bispecific regimens separately, and post-discontinuation benefit, and a threshold analysis identified the bispecific treatment duration at which cost per standardized progression-free month equaled that of cilta-cel. The drug-acquisition numerator was defined identically across arms using product cost, including cilta-cel; the loaded one-time cost was used only for the cumulative comparison. Severe immune-related adverse-event costs (grade greater than 2 CRS and neurotoxicity) were costed separately, and immunoglobulin replacement, incurred by all arms, was addressed narratively.

## Results

### Included trial characteristics

**Fig. 2:**
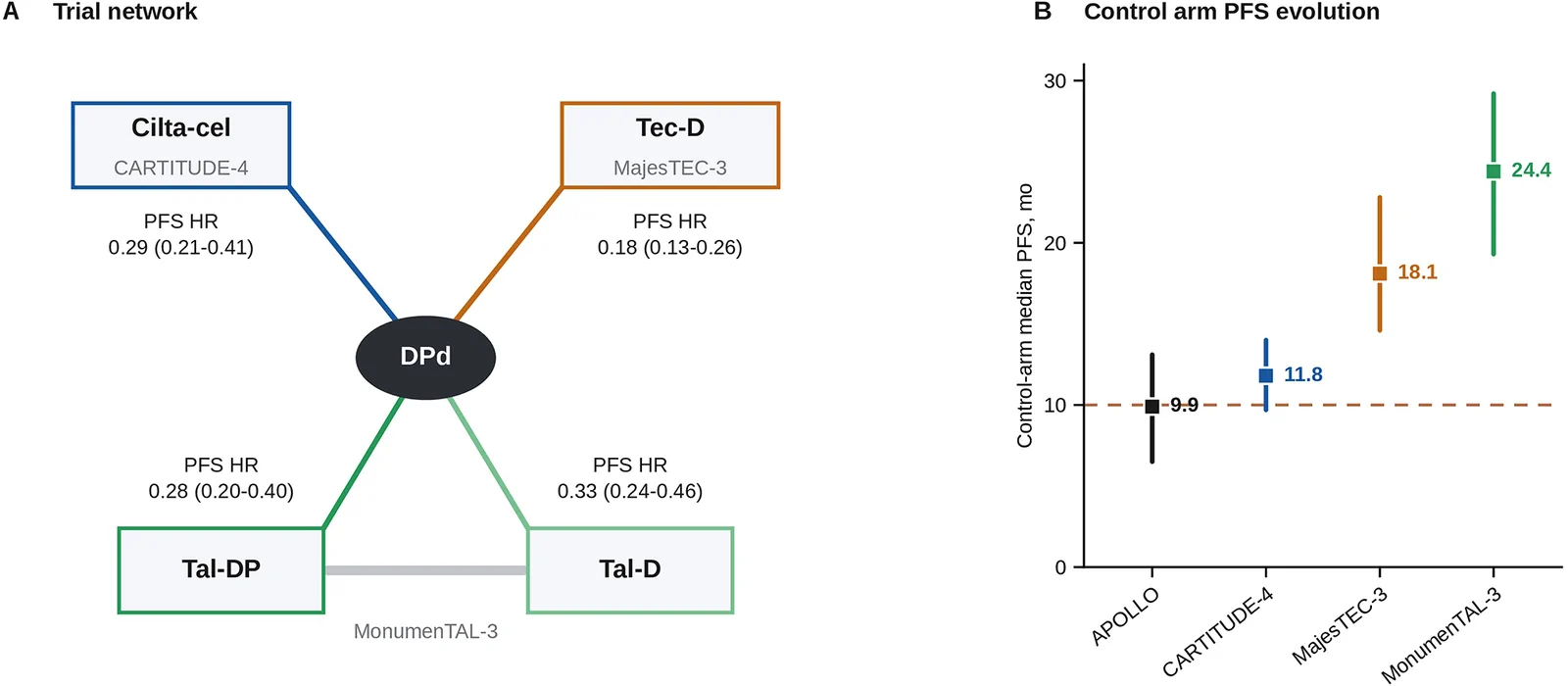
Network and control-arm evolution. **A**, Network of the 4 included trials anchored on the shared daratumumab-pomalidomide-dexamethasone (DPd)-type control. Nodes denote treatments and edges denote randomized comparisons, each derived from a single trial, with edge labels showing PFS hazard ratio versus control. **B**, Control-arm median PFS across trials

Four pivotal randomized phase 3 trials comprising 2,174 patients met eligibility and formed the DPd-anchored network (Fig. 2A): CARTITUDE-4 (cilta-cel) [23, 24], MajesTEC-3 (teclistamab-daratumumab) [25], MonumenTAL-3 (talquetamab-daratumumab with or without pomalidomide) [26], and APOLLO (DPd, providing the registrational anchor) [27, 28]. Trial characteristics of included and key excluded trials, and anchored estimates are summarized in Table 1 and Supplemental Table 1.

**Table 1:** Characteristics of studies included in the quantitative network meta-analysis and of studies reported qualitatively only

Trial (agent)	Control	Number	Prior lines (%)	Refractory to len / anti-CD38, %	Median follow-up, months	PFS, HR (95% CI)	OS, HR (95% CI)
CARTITUDE-4 (cilta-cel)	DPd or PVd	208 / 211	1 (33) 2 (40) 3 (27)	100 / 24	33.6	0.29 (0.22–0.39)	0.55 (0.39–0.79)
MajesTEC-3 (Tec-dara)	DPd or DVd	291 / 296	1 (37) 2 (46) 3 (17)	84 / 0	34.5	0.17 (0.12–0.23)	0.46 (0.32–0.65)
MonumenTAL-3 (Tal-DP)	DPd	287 / 290	1 (39) 2–3 (52) > 3 (9)	85 / 0	24.6	0.28 (0.20–0.40)	0.47 (0.30–0.73)
MonumenTAL-3 (Tal-D)	DPd	287 / 290	1 (37) 2–3 (53) > 3 (10)	85 / 0	24.6	0.33 (0.24–0.46)	0.51 (0.33–0.78)
APOLLO (DPd anchor)	Pd	151 / 153	2 (range 2–3)	79 / 0	39.6	12.4 (8.3–19.3)^*^	0.82 (0.61–1.11)
**Not included in quantitative analysis**							
MajesTEC-9 (Tec mono)	PVd or Kd	296 / 297	1 (22) 2–3 (78)	79 / 85	17.3	0.29 (0.23–0.38)	0.60 (0.43–0.83)
MagnetisMM-5 (Elra mono)	DPd	497^†^	NR	NR^‡^	NR	NR (topline)^§^	NR

Abbreviations: CI, confidence interval; ctrl, control; cilta-cel, ciltacabtagene autoleucel; Tec, teclistamab; Tal, talquetamab; Elra, elranatamab; DPd, daratumumab-pomalidomide-dexamethasone; DVd, daratumumab-bortezomib-dexamethasone; HR, hazard ratio; Kd, carfilzomib-dexamethasone; NR, not reported; OS, overall survival; Pd, pomalidomide-dexamethasone; PFS, progression-free survival; PVd, pomalidomide-bortezomib-dexamethasone

* Value shown is median PFS in months, not a hazard ratio; APOLLO defines the DPd anchor rather than an experimental contrast

† MagnetisMM-5 randomized 497 patients 1:1:1 to elranatamab monotherapy, elranatamab plus daratumumab, or DPd; per-arm numbers for the elranatamab-versus-DPd comparison were not disclosed

‡ Prior lenalidomide and a proteasome inhibitor were required; anti-CD38-refractory patients were ineligible for the DPd comparator, and refractoriness proportions were not reported

§ The primary PFS endpoint was met (elranatamab superior to DPd by blinded independent central review), but the hazard ratio, its interval, and median PFS were not disclosed; overall survival was immature

All four included trials for quantitative analysis were open-label, yielding an overall risk of bias rating of some concerns for the PFS outcome, driven by the deviations-from-intended-intervention and outcome-measurement domains, the latter was mitigated where PFS was assessed by an independent review committee and more concerning where investigator-assessed (Supplemental Fig. 1). For OS, the more objective outcome, risk of bias ranged from low to some concerns.

### Heterogeneity of the DPd control

The DPd-based control performed differently between trials (Fig. 2B and Supplemental Table 2). Control-arm median PFS spanned a 2.5-fold range, from 9.9 months in the APOLLO lenalidomide-refractory subgroup and 11.8 months in CARTITUDE-4 to 18.1 months in MajesTEC-3 and 24.4 months in MonumenTAL-3 (I-squared 91%; tau-squared 0.143). The two shortest medians occurred in the two populations that were entirely lenalidomide-refractory, and CARTITUDE-4 was the only trial admitting anti-CD38-refractory disease (24%). The trials further differed in the number of prior lines, prior anti-CD38 exposure, enrollment period, and control-regimen composition (Supplemental Table 3). With four control arms these contributors cannot be separated, and the dispersion is best regarded as reflecting several co-occurring differences in the enrolled populations.

### Survival

**Fig. 3:**
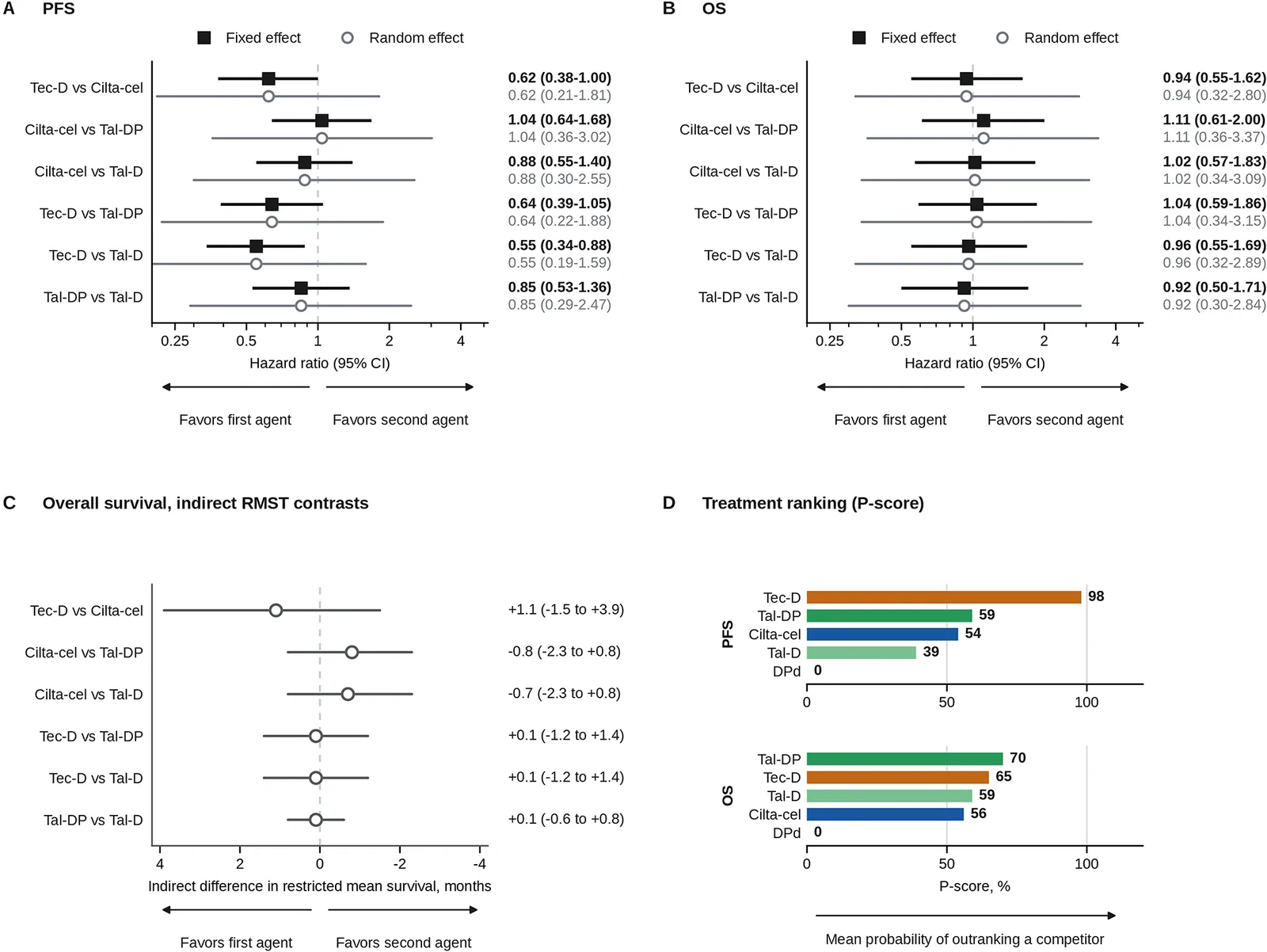
Comparative efficacy. **A**, Forest plot of pairwise indirect PFS contrasts under fixed-effect and random-effects models, showing intention-to-treat estimates. **B**, Forest plot of OS contrasts under both models. **C**, Restricted mean survival time (RMST) differences versus control at 24 months, extended to 36 months where follow-up permitted. **D**, Treatment ranking by frequentist network meta-analysis for PFS and OS

No comparison demonstrated robust separation among the four regimens for PFS. Under a fixed-effect model (Fig. 3A), which assumes the four DPd control arms are exchangeable, only 1 of 6 contrasts reached significance: teclistamab-daratumumab versus cilta-cel and versus talquetamab-daratumumab-pomalidomide approached but did not cross unity (0.62 [95% CrI, 0.38-1.00] and 0.64 [0.39–1.05], respectively), whereas teclistamab-daratumumab versus talquetamab-daratumumab remained significant (0.55 [0.34–0.88]). Once between-trial variability was carried through in a random-effects model, no contrast remained significant: every interval widened across 1 (teclistamab-daratumumab versus cilta-cel 0.62 [0.21–1.81]; versus talquetamab-daratumumab-pomalidomide 0.64 [0.22–1.88]; versus talquetamab-daratumumab 0.55 [0.19–1.59]), and the probability that any one agent truly outperformed another remained modest (0.87 that teclistamab-daratumumab exceeds cilta-cel; 0.85 and 0.91 that it exceeds talquetamab-daratumumab-pomalidomide and talquetamab-daratumumab, respectively; Supplemental Tables 4 and Supplemental Fig. 2–4). Conclusions were unchanged across heterogeneity priors: under the narrowest prior (scale 0.16), no PFS or OS contrast excluded 1 (Supplemental Fig. 5), and the only fixed-effect separation, teclistamab-daratumumab versus talquetamab-daratumumab, no longer excluded 1 at a tau of 0.14, a value with a prior probability of 0.33 under the primary prior.

The teclistamab-daratumumab versus cilta-cel contrast warranted further scrutiny because anti-CD38-refractory disease, the trial-level difference most clearly confined to a single trial, was present in approximately 24% of CARTITUDE-4 patients and excluded from the comparator trials. Within CARTITUDE-4 the standard-of-care control declines markedly in anti-CD38-refractory patients, so their inclusion lowered rather than inflated cilta-cel’s intention-to-treat hazard ratio. To test whether this imbalance drove the apparent separation, we repeated the analysis using the published subgroups without prior anti-CD38 exposure for CARTITUDE-4 and MonumenTAL-3, and the DPd-stratum estimate for MajesTEC-3, in which only 5.3% of patients had prior anti-CD38 exposure. Restricting the network in this way strengthened the teclistamab-daratumumab versus cilta-cel contrast to a fixed-effect hazard ratio of 0.58 (95% CrI, 0.36 to 0.95), with a posterior probability of a lower PFS hazard for teclistamab-daratumumab of 0.89; 2 of 6 contrasts excluded 1 under fixed effects, compared with 1 of 6 in the primary analysis (Supplemental Tables 5 and Supplemental Fig. 6). This indicates that inclusion of anti-CD38-refractory patients attenuated rather than created the apparent teclistamab-daratumumab advantage. As in the primary analysis, no contrast excluded 1 once between-trial heterogeneity was admitted, so the qualitative conclusion was unchanged.

Overall survival was concordant across regimens, and no pairwise OS contrast reached significance under the fixed-effect or the random-effects model (Fig. 3B). Anchored OS hazard ratios were 0.52 for cilta-cel, 0.49 for teclistamab-daratumumab, 0.47 for talquetamab-daratumumab-pomalidomide, and 0.51 for talquetamab-daratumumab 0.51. No pairwise indirect contrast approached significance under either model. The teclistamab-daratumumab versus cilta-cel comparison, the OS contrast of greatest clinical interest, gave an indirect hazard ratio of 0.94 (95% CrI, 0.55–1.62), an interval compatible with a substantial advantage for either agent. Because the OS curves crossed in several trials, an analysis assuming proportional hazards is incomplete; we therefore confirmed concordance with a proportional-hazards-free analysis of restricted mean survival.

Similarity could not be inferred: under the primary model, the posterior probability that a hazard ratio lay within 0.80 to 1.25 did not exceed 0.43 for any between-trial PFS contrast or 0.40 for any between-trial OS contrast (Supplemental Table 4). With intention-to-treat estimates, which included PVd- and DVd-treated controls, 3 of 6 fixed-effect contrasts excluded 1 (teclistamab-daratumumab versus cilta-cel, 0.59 [95% CrI, 0.38–0.90]; versus talquetamab-daratumumab-pomalidomide, 0.61 [0.38–0.98]; versus talquetamab-daratumumab, 0.52 [0.33–0.82]), whereas none did under random effects. Overall survival contrasts remained non-significant under both models (Supplementary Table 4).

Within each trial, restricted mean survival differences versus the shared control at 24 months were small and similar (cilta-cel + 0.7 months [95% CI, -0.6 to + 2.0]; teclistamab-daratumumab + 1.6 [+ 0.6 to + 2.5]; talquetamab-daratumumab-pomalidomide + 1.5 [+ 0.6 to + 2.3]; talquetamab-daratumumab + 1.4 [+ 0.6 to + 2.3]). The cilta-cel difference was the only one to include zero at 24 months, because its survival advantage accrued later (Supplemental Fig. 7). Extending the horizon to 36 months, supported by CARTITUDE-4 and MajesTEC-3 follow-up, increased the cilta-cel within-trial difference to + 2.3 months (95% CI, + 0.1 to + 4.5), an interval whose lower bound approaches zero. The between-regimen teclistamab-daratumumab versus cilta-cel indirect contrast remained non-significant at 36 months (+ 1.1 months [95% CI, -1.6 to + 3.9]; Fig. 3C).

Treatment rankings reinforced rather than resolved this picture (Fig. 3D). Teclistamab-daratumumab ranked first for PFS (P-score 98%), but the ordering reshuffled entirely for OS, where the four active regimens compressed into a narrow 56% to 70% band, each value approximating the roughly 62% a treatment obtains by outranking only the DPd anchor while remaining indistinguishable from every competitor. The apparent PFS hierarchy is therefore a property of a single endpoint and does not carry to OS. Because the P-score reflects the mean probability of outranking a competing treatment, including the DPd anchor, rather than the probability of being the best treatment, it was also computed among the four active regimens only: the PFS P-score of teclistamab-daratumumab was unchanged (98%), and OS P-scores ranged from 42% to 60%.

### Safety and second primary malignancies

Because safety endpoints were not amenable to anchored indirect comparison, the toxicity profiles are summarized descriptively, recognizing that adverse-event ascertainment, supportive-care guidance, and follow-up duration differed across trials (Supplemental Table 6). With cilta-cel, CRS occurred in 75% of treated patients but was grade 3 in approximately 1% with no grade 4 or 5 events, and ICANS occurred in about 5%, all grade 1 or 2. With the bispecific-antibody combinations, CRS was confined to the step-up dosing period and remained grade 1 or 2 in essentially all patients: in MajesTEC-3 the CRS rate was 60% (grade 1/2 only) and ICANS was 1%, while in MonumenTAL-3 the talquetamab arms showed CRS incidences of 68% (talquetamab-daratumumab-pomalidomide) and 58% (talquetamab-daratumumab), largely grade 1–2 with rare grade 3 or higher events, and ICANS in 3% and 2% of recipients, respectively, including three resolved grade 3 events in the triplet arm. Cilta-cel was further distinguished by a small but characteristic burden of delayed CAR-T-specific neurotoxicity, comprising treatment-emergent parkinsonism in approximately 1% together with cranial-nerve palsies, not observed with the bispecifics, whereas the talquetamab regimens carried GPRC5D-specific dysgeusia, skin and nail toxicity, ataxia and weight loss, predominantly low grade.

Second primary malignancies (SPMs) did not distinguish the BCMA-directed regimens (Supplemental Fig. 8). Total SPM incidence was 13.0% for cilta-cel and 12.4% for teclistamab-daratumumab at comparable median follow-up (33.6 and 34.5 months), and in both trials exceeded the respective control arm (11.5% and 8.6%). The talquetamab combinations showed numerically lower totals (7.6% and 3.6%, respectively), but at a shorter median follow-up of 24.6 months, precluding direct cross-trial comparison of absolute rates. One consistent finding was a higher hematologic SPM rate in each experimental arm than in its control (cilta-cel 3.4% vs. 0.5%; teclistamab-daratumumab 2.8% vs. 1.4%; talquetamab-daratumumab-pomalidomide 2.5% vs. 0%).

### Cost consequences

**Fig. 4:**
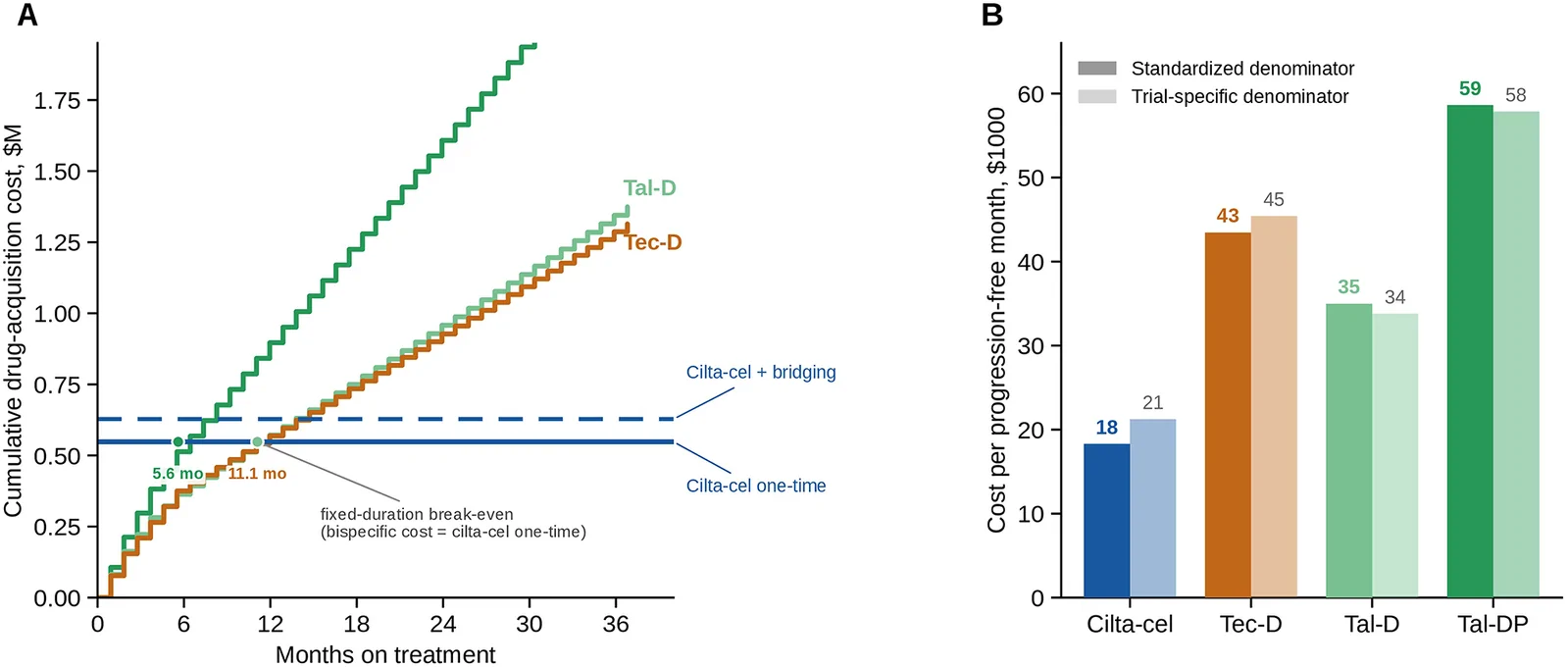
Costs and scenarios. **A**, Cumulative drug-acquisition cost over time. **B**, Cost per progression-free month by different denominators

The regimens differed structurally in drug-acquisition cost. Because cilta-cel is administered once, its drug-acquisition cost is fixed at $547,940, whereas the cost of each continuously dosed regimen accrues throughout treatment and surpasses this one-time cost within the first year, at approximately 11 months for teclistamab-daratumumab and talquetamab-daratumumab and 6 months for talquetamab-daratumumab-pomalidomide (Fig. 4A and Supplemental Table 7). Median treatment durations of 21 to 32 months exceed these crossover points two- to fourfold, so every continuously dosed regimen accumulated more drug cost than cilta-cel over a realistic course; a talquetamab bridging cycle raised the cilta-cel benchmark to $622,948 and shifted each crossover by less than three months. At median treatment duration, the cilta-cel one-time cost was 47% of the cumulative teclistamab-daratumumab cost ($1,176,237), and severe immune-related adverse-event management costs were negligible across regimens ($269 to $1,290 per patient). With the population-standardized 30-month restricted mean PFS denominator, cost per progression-free month was $18,300 for cilta-cel, $43,452 for teclistamab-daratumumab, $34,976 for talquetamab-daratumumab, and $58,629 for talquetamab-daratumumab-pomalidomide, at observed median treatment durations (Fig. 4B). The standardized denominator narrowed the gap between cilta-cel and teclistamab-daratumumab relative to trial-specific denominators ($21,233 versus $45,415 per month), because the longer control-arm PFS in MajesTEC-3 inflated the trial-specific benefit estimate. Cost per standardized progression-free month of teclistamab-daratumumab equaled that of cilta-cel at a treatment duration of 9.7 months, and of talquetamab-daratumumab and talquetamab-daratumumab-pomalidomide at 8.7 and 5.1 months, respectively; a 40% reduction in bispecific prices lowered these to $26,071, $20,986, and $35,177, respectively (Supplemental Fig. 9).

## Discussion

In this network meta-analysis of T-cell-redirecting regimens in early relapsed MM, the randomized evidence did not establish an efficacy hierarchy. Point estimates for PFS favored teclistamab-daratumumab, but this separation depended on assuming exchangeable trials and disappeared once modest between-trial heterogeneity was allowed. Overall survival did not separate under any model. The intervals remain compatible with clinically meaningful differences in either direction.

Random-effects intervals depend on an externally specified heterogeneity prior; however, any separation required heterogeneity close to zero, which the observed control-arm dispersion makes implausible.The governing observation was the behavior of the shared control. Although all anchored trials used a predominantly daratumumab-pomalidomide-based control, control-arm median PFS differed 2.5-fold. Because control-arm performance indexes the enrolled population rather than any experimental regimen, this dispersion indicates that population differences, including lenalidomide and anti-CD38 refractoriness, number of prior lines, and enrollment period, contribute substantially to cross-trial variation. Any indirect contrast referred to this anchor is shaped by these differences as well as by the regimens compared.

The modalities are not symmetric with respect to sequence. Antigen escape through BCMA loss occurs in up to half of patients relapsing after BCMA-directed bispecific antibodies but appears less common with CAR T cells [29, 30]. In addition, tonic engagement required for continuous bispecific dosing can impact T-cell fitness on which a subsequent autologous CAR product depends [31], whereas a single cilta-cel infusion expands transiently and then contracts, after which endogenous T-cell function can recover. These asymmetries make the order of administration biologically non-neutral, and the available non-randomized evidence that CAR T cells followed by bispecific antibodies outperform the reverse sequence is consistent with these biological considerations, though hypothesis-generating and not tested in early relapse [11, 12].

This biology must be weighed against the opposing practical asymmetry: bispecific antibodies are available off-the-shelf and can be started immediately, whereas CAR T-cell therapy requires substantial amount of planning and resources surrounding apheresis, manufacturing, and bridging [32, 33]. This matters most for rapidly progressive disease and for patient and societal environments with differential cell-therapy access [34].

The toxicity profiles differ across modalities and warrant individualized discussion, although their overall burden cannot be reliably compared across trials. Cilta-cel carries a small but characteristic burden of delayed, occasionally irreversible neurotoxicity, with immune effector cell-associated enterocolitis and rare CAR-derived T-cell malignancy [4, 35]. In contrast, the GPRC5D-directed agents carry dysgeusia, skin and nail toxicity, weight loss and cerebellar disorders [36]. Continuous T-cell engagement, particularly with BCMA-directed bispecifics, imposes sustained hypogammaglobulinemia and a high incidence of severe infection. Each profile is increasingly modifiable, the cilta-cel movement disorder through patient selection, bridging, and pre-emptive management, and bispecific-associated infection through immunoglobulin replacement, prophylaxis, and step-up optimization, so the relevant question is which toxicity profile a given patient is best positioned to tolerate. Much of the continuous-dosing toxicity and cost burden may also be mitigated as practice shifts toward fixed-duration bispecific administration, an approach being evaluated in currently-enrolling trials and already adopted by many clinicians. SPM did not distinguish the BCMA-directed regimens, with a modestly higher hematologic rate in each experimental arm than its control while the lower talquetamab totals were observed at shorter follow-up. More detailed prospective analysis of these patients with longer follow-up is needed to determine potential differences in oncogenic risk between cellular therapy and T-cell engagers. In the absence of an established efficacy hierarchy, toxicity, sequencing, logistics, patient preference, and cost will inform treatment selection, although none of these dimensions has been compared in randomized fashion, and safety data differ across trials in follow-up, exposure, and ascertainment.

Drug-acquisition costs differed structurally between modalities. The drug-acquisition cost of a single cilta-cel infusion ($465,000) was 40% of the cumulative cost of teclistamab-daratumumab at median treatment duration. With a population-standardized denominator, cost per progression-free month was lowest for cilta-cel, but this reflects a one-time cost set against a modeled progression-free interval and is confined to US drug-acquisition costs. It does not capture broader resource use or health outcomes and does not constitute a cost-effectiveness analysis. A single-infusion product whose cost is incurred once and whose benefit does not depend on continued administration is economically distinct from an indefinitely dosed agent in a way that interacts with the local care setting a distinction that grows more salient as these therapies move into earlier lines, larger populations, as well as potential for academic or point-of-care production [37, 38]. However, these acquisition-cost comparisons do not capture the uncertain and potentially substantial costs of managing severe toxicities, including immune effector cell-associated enterocolitis, high-grade CRS or hemophagocytosis, neurotoxicity, and prolonged cytopenia, which could narrow the apparent cost advantage of a one-time therapy. With a population-standardized denominator that applies each regimen’s anchored hazard ratio to a common reference DPd curve, cost per progression-free month was lowest for cilta-cel, but this reflects a one-time cost set against a modeled progression-free interval and does not constitute a cost-effectiveness analysis. These acquisition-cost comparisons reflect US wholesale prices and may not apply to other health systems, where net prices differ and pomalidomide is available as a generic.

These considerations are becoming more consequential as both modalities move into frontline, where the eligible populations enlarge and increasingly overlap and the decision shifts from whether to use a T-cell-redirecting therapy to which one, and in what order. That trajectory is already visible: cilta-cel, teclistamab, and talquetamab are being tested in newly diagnosed disease and in high-risk smoldering myeloma [39–41].

This study has several limitations. The analysis is an anchored indirect comparison, not a head-to-head trial, and its validity rests on transitivity and the absence of effect-modifier imbalance, the very conditions that the demonstrated control-arm heterogeneity shows to be strained. We reported fixed- and random-effects estimates side by side precisely to expose the dependence of any separation on this assumption. The network formed a star, with every comparison made via the shared control and no direct comparison between experimental regimens; there are therefore no closed loops with which to test consistency, and transitivity must be assumed. With a single trial per edge, the Bayesian and frequentist models reduce to pairwise anchored (Bucher-type) indirect comparisons, so the multiple frameworks serve as robustness checks on the same contrasts rather than as independent evidence. Each network edge derived from a single trial, so between-trial heterogeneity could not be estimated empirically, and for trials reporting numbers-at-risk only, patient-level data were reconstructed from published curves, which may introduce reading error. Eligibility also differed in the number of permitted prior lines of therapy: MonumenTAL-3 enrolled patients beyond three prior lines, whereas the other trials were restricted to one to three, adding to the population differences across the network. In addition, approximately 15% of patients assigned to cilta-cel in CARTITUDE-4 did not reach CAR T-cell infusion; although the intention-to-treat analysis retains these patients, differences across trials in how non-infused patients are captured complicate the cross-trial comparison. The requirement for a shared daratumumab-pomalidomide anchor and for extractable randomized data also constrained the network. A small proportion of control patients received PVd (CARTITUDE-4) or DVd (MajesTEC-3) rather than DPd, which may have contributed to differences in control-arm performance across trials. It excluded MajesTEC-9, in which teclistamab monotherapy was compared against a pomalidomide- or carfilzomib-based control in a more heavily anti-CD38-refractory population and reported comparable efficacy, because its different comparator placed it outside the anchor [42]. It likewise excluded MagnetisMM-5, which shares the daratumumab-pomalidomide anchor and would have added a fifth agent, because its phase 3 results have so far been reported only in summary form without the published time-to-event data required for curve reconstruction. This trial should be incorporated as their full data mature, for which our results may represent a benchmark for evaluating potential comparative efficacy and value. Safety, sequencing, and cost endpoints were assessed descriptively and are not anchored comparisons, and follow-up differed across trials, with the talquetamab combinations assessed earliest. Drug-acquisition costs used wholesale acquisition cost without adjustment to present value. The use of list rather than net prices and the absence of present-value adjustment both inflate the cost of continuously-dosed regimens, so the modeled gap likely overstates cilta-cel’s advantage. These analyses are accordingly hypothesis-generating and are intended to inform cross-trial interpretation rather than to substitute for an individual-patient-data meta-analysis or a randomized comparison.

## Conclusions

Taken together, the available randomized evidence does not establish an efficacy hierarchy among cilta-cel, teclistamab-daratumumab, and the talquetamab combinations in early relapsed MM, nor does it demonstrate equivalence, unlike in later lines of therapy where CAR T cells showed improved outcomes compared with bispecific antibodies. Until comparative data exist, treatment selection cannot rest on efficacy data alone and will be informed by patient preference and patient- and disease-related factors, including side-effect profiles, treatment sequence, access, and cost.

## Supplementary Information

Below is the link to the electronic supplementary material.

Supplementary Material 1

## Data Availability

No datasets were generated or analysed during the current study.
