# Supplementary material for: T-cell-redirecting therapies in early relapsed multiple myeloma: a systematic review and network meta-analysis
Source: J Hematol Oncol. 2026 Oct 7;19:83. doi: 10.1186/s13045-026-01850-2 (PMC13644290; doi:10.1186/s13045-026-01850-2)
Supplement: Supplementary file 1 [file 13045_2026_1850_MOESM1_ESM.docx]

**Online Supplemental Content**

**T-cell-redirecting therapies in early relapsed multiple myeloma:
a systematic review and network meta-analysis**

Merz M, et al.

**Contents**

1. Supplemental Methods

2. Supplemental Results

3. Supplemental Tables (Tables S1-S7)

4. Supplemental Figures (Figures S1-S9)

**1. Supplementary Methods**

**1.1 Search strategy**

("Multiple Myeloma"[Mesh] OR multiple myeloma[tiab] OR plasma cell myeloma[tiab] OR myeloma[tiab] OR RRMM[tiab]) AND ("Receptors, Chimeric Antigen"[Mesh] OR "Immunotherapy, Adoptive"[Mesh] OR "Antibodies, Bispecific"[Mesh] OR chimeric antigen receptor[tiab] OR CAR-T[tiab] OR "CAR T"[tiab] OR CART[tiab] OR bispecific[tiab] OR "T-cell engager"[tiab] OR "T cell engager"[tiab] OR BiTE[tiab] OR "T-cell redirecting"[tiab] OR "T cell redirecting"[tiab] OR "T-cell-redirecting"[tiab] OR BCMA[tiab] OR "B-cell maturation antigen"[tiab] OR TNFRSF17[tiab] OR GPRC5D[tiab] OR ciltacabtagene[tiab] OR cilta-cel[tiab] OR Carvykti[tiab] OR "LCAR-B38M"[tiab] OR "JNJ-4528"[tiab] OR idecabtagene[tiab] OR ide-cel[tiab] OR Abecma[tiab] OR "bb2121"[tiab] OR teclistamab[tiab] OR Tecvayli[tiab] OR "JNJ-64007957"[tiab] OR talquetamab[tiab] OR Talvey[tiab] OR "JNJ-64407564"[tiab] OR elranatamab[tiab] OR Elrexfio[tiab] OR "PF-06863135"[tiab] OR linvoseltamab[tiab] OR "REGN5458"[tiab] OR alnuctamab[tiab] OR "ABBV-383"[tiab]) AND (randomized controlled trial[pt] OR controlled clinical trial[pt] OR randomized[tiab] OR placebo[tiab] OR "clinical trials as topic"[Mesh:noexp] OR randomly[tiab] OR trial[ti]) NOT (animals[mh] NOT humans[mh])

**1.2 Data inputs and provenance**

All inputs were transcribed from the primary trial reports. No individual patient data were used. Three input sets enter the analysis, each provided as a table rather than in the text: the anchored hazard ratios for each agent versus the shared daratumumab-pomalidomide-dexamethasone (DPd)-type control, the control-arm median progression-free survival in the lenalidomide-refractory frame, and the drug-acquisition cost parameters. The standard error of each log-hazard-ratio was derived from its 95% confidence interval as (ln[upper] - ln[lower]) / 3.92. The time-to-event inputs were numbers-at-risk tables and published Kaplan-Meier curves, from which pseudo-patient data were reconstructed.

**1.3 Reconstruction of patient-level survival data (Guyot algorithm)**

Pseudo-patient time-to-event data were reconstructed from the published Kaplan-Meier curves and the accompanying numbers-at-risk tables using the Guyot algorithm, which inverts the product-limit relationship between curve height, numbers at risk, and interval events to recover a patient-level dataset consistent with the reported curve. Two variants were applied according to the information each trial disclosed. Where cumulative censoring counts were reported (CARTITUDE-4, APOLLO), the per-interval events and censorings were recovered exactly from the change in numbers at risk net of reported censoring. Where only numbers at risk were available (MajesTEC-3, MonumenTAL-3), the number of events in each interval was inferred from the decrement in the survival curve between adjacent reading points, with censorings assigned as the residual change in the risk set. Within each interval, event and censoring times were distributed uniformly. The exact variant reproduced the reported event totals exactly, recovering in CARTITUDE-4 the 89 cilta-cel and 153 standard-of-care progression-free-survival events, which provides a positive control on the procedure. Estimates from trials reconstructed by the survival-height variant are flagged as such.

**1.4 Survival estimation: Kaplan-Meier, median, and restricted mean survival time**

From the reconstructed data, survival was estimated by the product-limit (Kaplan-Meier) estimator. The median survival is the first time at which the estimated survival function falls to 0.5. The restricted mean survival time (RMST) to a horizon τ is the area under the survival curve from time zero to τ, computed as a step-function integral; it summarizes the average event-free time over a fixed window and, unlike the hazard ratio, does not assume proportional hazards.

**1.5 Heterogeneity of the shared control arm (DerSimonian-Laird)**

Because the validity of any anchored comparison rests on the exchangeability of the shared control, between-trial heterogeneity of the DPd-type control arm was quantified explicitly by a random-effects synthesis of the log control-arm median PFS. With per-trial estimates yᵢ and variances sᵢ² (the latter obtained from the reported confidence intervals on the log scale), Cochran’s Q with weights. The between-study variance was τ², and the inconsistency index I-squared.

**1.6 Anchored indirect comparison: Bayesian and frequentist network meta-analysis**

Because no head-to-head trials exist, treatments were compared indirectly through their shared DPd-type anchor using the Bucher relation: the log-hazard-ratio contrast between two experimental agents equals the difference of their anchored log-hazard-ratios, with variance equal to the sum of the anchored variances. Because each network edge derives from a single trial, the network is a star with no closed loops, so the between-trial heterogeneity standard deviation τ cannot be estimated from the data; it was assigned a weakly informative half-normal prior (standard deviation 0.32), consistent with empirical distributions of heterogeneity for time-to-event pharmacological comparisons. Two models were reported for every contrast: a fixed-effect model (τ = 0) and a random-effects model that propagates the prior. Posterior summaries were obtained exactly rather than by simulation. Conditional on tau the contrast is normally distributed, and tau carries a half-normal prior, so the marginal is a scale mixture of normals; its distribution function was evaluated by quadrature over the prior and the quantiles obtained by root-finding. The random-effects estimates and the posterior probability P(HR<1) are therefore deterministic and reproduce exactly in any implementation.

To confirm that the conclusions did not depend on the Bayesian framework or its heterogeneity prior, the same network was re-estimated in a frequentist framework using the graph-theoretical (netmeta) approach of Ruecker et al. with DerSimonian-Laird heterogeneity. Because the network is a star with a single study per edge, no within-network heterogeneity is estimable and the frequentist random-effects model reduces to the fixed-effect model. The frequentist pairwise hazard ratios therefore coincide with the Bayesian fixed-effect estimates. Treatments were ranked by P-score, the frequentist analogue of the surface under the cumulative ranking curve, defined for each treatment as the mean over competitors of the probability of superiority derived from the point estimate and its standard error. The P-score is the mean probability that a treatment outranks a competing treatment and is not the probability of being the best treatment.

**1.7 Overall survival RMST layer and bootstrap**

To avoid relying on the proportional-hazards assumption for overall survival, which is questionable where survival curves cross, overall survival was additionally compared as the difference in restricted mean survival time at a common 24-month horizon, with a sensitivity analysis extending the horizon to 36 months where follow-up permitted. The reconstructed arms were used directly; confidence intervals were obtained by nonparametric bootstrap, resampling patients within each arm with replacement (20,000 resamples) under a fixed seed for reproducibility.

**1.8 Harmonization to subgroups without prior anti-CD38 exposure**

The cilta-cel versus teclistamab-daratumumab contrast is the only edge affected by an effect modifier distributed unevenly across the network: anti-CD38-refractory disease, present in approximately 24% of CARTITUDE-4 but excluded from the comparator trials. To test whether this imbalance drove the apparent separation, each trial was restricted to its anti-CD38-naïve stratum and the network re-estimated. Because the comparator trials’ naïve strata coincide with their full populations, only the cilta-cel edge changes: the cilta-cel hazard ratio versus DPd rises from 0.29 (intention-to-treat) to 0.31 (naïve), reflecting removal from the cilta-cel arm of the most refractory patients, whose standard-of-care control collapses. The harmonized network was summarized under both fixed- and random-effects models.

**1.9 Cost-consequence model**

Drug-acquisition cost was modeled from US wholesale acquisition cost for an 80-kg patient without wastage. Ciltacabtagene autoleucel was modeled as a one-time loaded cost comprising product, apheresis, lymphodepletion, bridging, and administration; the bispecific regimens were accrued cycle-by-cycle per label dosing (daratumumab weekly, then biweekly, then monthly; teclistamab and talquetamab per their schedules; pomalidomide per cycle for the triplet). The outcome was drug-acquisition cost per progression-free month. The primary denominator was a population-standardized 30-month restricted mean PFS, obtained by applying each regimen's anchored hazard ratio to a common reference DPd curve derived from the pooled reconstructed control arms, so that between-trial differences in absolute PFS did not enter the cost ratio; each trial's reconstructed 30-month restricted mean PFS served as a secondary, trial-specific denominator. Deterministic sensitivity analyses varied treatment duration (12, 18, and 24 months and each trial's observed median treatment duration), acquisition cost of the CAR T-cell and bispecific regimens separately (reductions of 20% and 40% as proxies for net prices), and post-discontinuation benefit (PFS benefit maintained versus reverting to the control hazard after treatment discontinuation, the maintained assumption being the one most favorable to the bispecific regimens). A threshold analysis identified the bispecific treatment duration at which cost per standardized progression-free month equaled that of cilta-cel. Severe immune-effector adverse-event management costs (grade greater than 2 cytokine release syndrome and neurotoxicity) were costed separately and were non-differentiating across agents. This is a cost-consequence, not a cost-utility, analysis; no quality-adjusted life-years were modeled.

**1.10 Assumptions, approximations, and provenance flags**

**Reconstruction.** CARTITUDE-4 and APOLLO reconstructions are exact (cumulative censoring reported); MajesTEC-3 and MonumenTAL-3 used survival-height reads and are flagged as such. Reconstructed event totals matched the reported totals where verifiable.

**Network structure.** The network is a star with single-trial edges and no closed loops; consistency cannot be assessed empirically and τ is not estimable from the data, motivating the weakly informative prior and the parallel reporting of fixed- and random-effects models.

**Inference.** The primary analysis is Bayesian, reporting the posterior probability of superiority P(HR<1) in preference to a p-value. A frequentist network meta-analysis with P-score ranking is reported as a prespecified confirmatory analysis.

**Cost.** Costs are modeled wholesale acquisition costs without wastage or discounting; cumulative costs refer to observed median treatment duration.

**2. Supplementary Results**

**2.1 Reconstruction validation**

The exact reconstruction reproduced the reported progression-free-survival event totals in CARTITUDE-4 (89 cilta-cel and 153 standard-of-care events), and reproduced reported medians and landmark survival to within reading error across trials.

**2.2 Heterogeneity of the shared control**

Control-arm median PFS spanned a 2.5-fold range across trials, from 9.9 months (APOLLO) to 24.4 months (MonumenTAL-3). The DerSimonian-Laird synthesis yielded I² = 91% and τ² = 0.143, with a random-effects pooled control-arm median of 15.2 months (95% CI, 10.3-22.5). This quantifies the between-trial inconsistency that governs every anchored contrast.

**2.3 Fixed- versus random-effects contrasts**

Under the fixed-effect model, 1 of 6 progression-free-survival contrasts (teclistamab-daratumumab versus talquetamab-daratumumab) and 0 of 6 overall-survival contrasts excluded 1; under the random-effects model, 0 of 6 excluded 1 in either endpoint. Point estimates are identical across the two models because a single-trial edge carries no information with which to update the heterogeneity prior; the random-effects credible intervals are wider, reflecting honest uncertainty about between-trial heterogeneity.

**2.4 Harmonization to subgroups without prior anti-CD38 exposure**

Anti-CD38-refractory disease was the only candidate effect modifier clearly confined to a single trial: it was present in approximately 24% of CARTITUDE-4 patients and was an exclusion criterion in the comparator trials, which nevertheless enrolled anti-CD38-exposed patients (approximately 5% in MajesTEC-3 and 12% in MonumenTAL-3). The comparator trials were therefore not anti-CD38-naive, and the analysis is labeled accordingly. To test whether this imbalance drove the apparent teclistamab-daratumumab versus cilta-cel separation, the network was re-estimated using the published subgroups without prior anti-CD38 exposure for CARTITUDE-4 and MonumenTAL-3; for MajesTEC-3, in which only 5.3% of patients had prior anti-CD38 exposure and no exposure-restricted subgroup was reported, the daratumumab-pomalidomide-dexamethasone-stratum estimate was retained. Because the restriction changed only the CARTITUDE-4 input materially, the cilta-cel edges moved while the others were essentially unchanged: the cilta-cel hazard ratio versus DPd rose from 0.29 to 0.31, reflecting removal from the cilta-cel arm of the most refractory patients, whose standard-of-care control performed worst. The teclistamab-daratumumab versus cilta-cel contrast strengthened to a fixed-effect hazard ratio of 0.58 (95% CrI, 0.36 to 0.95), with the posterior probability that teclistamab-daratumumab improves progression-free survival rising to 0.89. Two of 6 contrasts thus excluded 1 under fixed effects, compared with 1 of 6 in the intention-to-treat analysis, indicating that inclusion of anti-CD38-refractory patients had attenuated rather than created the apparent teclistamab-daratumumab advantage over cilta-cel. All six contrasts again crossed 1 once between-trial heterogeneity was admitted, so the qualitative conclusion was unchanged.

**2.5 Frequentist confirmation and treatment ranking**

The frequentist network meta-analysis reproduced the Bayesian fixed-effect estimates, as expected for a single-study-per-edge star network, and reached concordant conclusions: the only pairwise contrast excluding 1 was teclistamab-daratumumab versus talquetamab-daratumumab on PFS, and no overall-survival contrast excluded 1. Treatment ranking by P-score was endpoint-dependent (Table S6). Teclistamab-daratumumab ranked first for PFS (P-score 98.2%), but the ordering reordered for overall survival, where the four active agents compressed into a 56% to 70% band, each value approximating the approximately 62% that a treatment obtains by outranking only the DPd anchor while remaining indistinguishable from the other active agents. The discordance of the ranking between endpoints, together with its compression for overall survival, corroborates the absence of a stable efficacy hierarchy.

**3. Supplementary Tables**

**Table S1. Anchored effect estimates: hazard ratios for each agent versus the shared DPd-type control.**

| **Trial (agent)** | **PFS HR (95% CI)** | **OS HR (95% CI)** |
| --- | --- | --- |
| CARTITUDE-4 (cilta-cel) | 0.29 (0.21-0.41) | 0.52 (0.35-0.77) |
| MajesTEC-3 (Tec-D) | 0.18 (0.13-0.26) | 0.49 (0.34-0.71) |
| MonumenTAL-3 (Tal-DP) | 0.28 (0.20-0.40) | 0.47 (0.30-0.73) |
| MonumenTAL-3 (Tal-D) | 0.33 (0.24-0.46) | 0.51 (0.33-0.78) |

**Table S2. Control-arm heterogeneity inputs: control-arm median PFS in the lenalidomide-refractory frame, with refractoriness composition, used in the random-effects synthesis.**

| **Trial** | **Median control PFS, mo**  **(95% CI)** | **Len-ref** | **Anti-CD38-ref** |
| --- | --- | --- | --- |
| MonumenTAL-3 | 24.4 (19.3-29.2) | 85% | Excluded |
| MajesTEC-3 | 18.1 (14.6-22.8) | 84% | Excluded |
| CARTITUDE-4 | 11.8 (9.7-14.0) | 100% | 24% included |
| APOLLO | 9.9 (6.5-13.1) | 100% | Excluded |

**Table S3. Trial-level characteristics and candidate effect modifiers.**

**Trial-level characteristics**

| **Charakteristik** | **CARTITUDE-4** | **MajesTEC-3** | **MonumenTAL-3** | **APOLLO** |
| --- | --- | --- | --- | --- |
| Enrollment period | Jul 2020 to Nov 2021 | Oct 2021 to Sep 2023 | Nov 2022 to Mar 2025 | Jun 2017 to Jun 2019 |
| Median follow-up, months | 33.6 | 34.5 | 24.6 | 16.9 (primary PFS analysis) |
| Permitted prior lines | 1 to 3 | 1 to 3 | 1 or more | 1 or more |
| Median prior lines (range) | 2 (1-3) | 2 (1-3) | 2 (1-8) | 2 (1-5) |
| One prior line, % | 32.5 | 37.8 | 38.3 | 11.2 |
| More than three prior lines, % | 0 | 0 | 9.5 | NR |
| Lenalidomide-refractory, % | 100 | 83.6 | 85.1 | 79.6 (benchmark subgroup: 100) |
| Proteasome inhibitor-refractory, % | Bortezomib 24.6; carfilzomib 22.9 | 37.6 | 42.9 | 48.0 |
| Prior anti-CD38 exposure, % | 25.8 | 5.3 | 11.9 | 0 (excluded) |
| Anti-CD38-refractory, % | 22.9 | 0 (excluded) | 0 (excluded) | 0 (excluded) |
| Triple-class-refractory, % | 15.0 | 0 | 0 | 0 |
| High-risk cytogenetics, % | 34.1 | 35.9 | 30.9 | NR |
| Cytogenetics missing or undetermined, % | 5.5 | 17.3 | 25.7 | NR |
| Soft-tissue plasmacytoma, % | 18.9 | 14.0 | 15.0 | NR |
| Extramedullary plasmacytoma, % | NR | 5.3 | 5.6 | NR |
| ISS stage III, % | 6.2 | 8.0 | 10.5 | 21.7 |
| ECOG performance status 2, % | 0.5 | 4.3 | 4.2 | 8.2 |
| PFS assessment | Central laboratory, computerized algorithm | Independent review committee | Independent review committee | Central, computerized algorithm |
| Control regimen, % | DPd 86.7; PVd 13.3 | DPd 90.9; DVd 9.1 | DPd 100 | DPd arm as benchmark (trial comparator Pd) |
| Control-arm median PFS in heterogeneity analysis, months | 11.8 | 18.1 | 24.4 | 9.9 (lenalidomide-refractory subgroup) |
| Assigned to experimental arm, not treated as assigned, % | 15.4 (not infused) | 2.7 | Tal-DP 3.8; Tal-D 4.5 | NA |
| Subsequent T-cell-redirecting therapy, control arm, % | CAR T 17.1; bispecific 32.7 | NR | 19.0 | NA |

**Within-trial evidence of effect modification for progression-free survival**

| **Candidate effect modifier** | **Trial** | **HR, first subgroup (95% CI)** | **HR, second subgroup (95% CI)** | **Ratio of HRs (95% CI)** |
| --- | --- | --- | --- | --- |
| Prior lines: 2-3 (MonumenTAL-3: 2 or more) vs 1 | CARTITUDE-4 | 0.26 (0.18-0.37) | 0.41 (0.25-0.67) | 0.63 (0.34-1.17) |
| Prior lines: 2-3 (MonumenTAL-3: 2 or more) vs 1 | MajesTEC-3 | 0.18 (0.12-0.27) | 0.14 (0.08-0.25) | 1.29 (0.64-2.59) |
| Prior lines: 2-3 (MonumenTAL-3: 2 or more) vs 1 | MonumenTAL-3, Tal-DP | 0.35 (0.24-0.52) | 0.19 (0.10-0.35) | 1.84 (0.88-3.85) |
| Prior lines: 2-3 (MonumenTAL-3: 2 or more) vs 1 | MonumenTAL-3, Tal-D | 0.43 (0.30-0.62) | 0.23 (0.13-0.40) | 1.87 (0.96-3.65) |
| High-risk vs standard-risk cytogenetics | CARTITUDE-4 | 0.26 (0.16-0.42) | 0.32 (0.18-0.59) | 0.81 (0.38-1.75) |
| High-risk vs standard-risk cytogenetics | MajesTEC-3 | 0.15 (0.09-0.25) | 0.16 (0.09-0.27) | 0.94 (0.44-1.98) |
| High-risk vs standard-risk cytogenetics | MonumenTAL-3, Tal-DP | 0.39 (0.24-0.65) | 0.22 (0.12-0.39) | 1.77 (0.82-3.84) |
| High-risk vs standard-risk cytogenetics | MonumenTAL-3, Tal-D | 0.36 (0.22-0.60) | 0.40 (0.24-0.65) | 0.90 (0.44-1.83) |
| Soft-tissue plasmacytoma: yes vs no | CARTITUDE-4 | 0.36 (0.20-0.66) | 0.28 (0.20-0.39) | 1.29 (0.65-2.55) |
| Soft-tissue plasmacytoma: yes vs no | MajesTEC-3 | 0.33 (0.17-0.63) | 0.13 (0.09-0.20) | 2.54 (1.18-5.47) |
| Soft-tissue plasmacytoma: yes vs no | MonumenTAL-3, Tal-DP | 0.41 (0.22-0.76) | 0.25 (0.17-0.37) | 1.64 (0.79-3.41) |
| Soft-tissue plasmacytoma: yes vs no | MonumenTAL-3, Tal-D | 0.34 (0.19-0.61) | 0.32 (0.22-0.46) | 1.06 (0.53-2.12) |
| ISS stage III vs I | CARTITUDE-4 | 0.41 (0.16-1.08) | 0.28 (0.19-0.41) | 1.46 (0.52-4.10) |
| ISS stage III vs I | MajesTEC-3 | 0.31 (0.12-0.79) | 0.12 (0.07-0.20) | 2.58 (0.88-7.60) |
| ISS stage III vs I | MonumenTAL-3, Tal-DP | 0.28 (0.12-0.67) | 0.26 (0.17-0.41) | 1.08 (0.41-2.83) |
| ISS stage III vs I | MonumenTAL-3, Tal-D | 0.31 (0.14-0.66) | 0.36 (0.24-0.54) | 0.86 (0.36-2.07) |
| Prior anti-CD38 exposure: yes vs no | CARTITUDE-4 | 0.24 (0.14-0.42) | 0.31 (0.22-0.44) | 0.77 (0.40-1.48) |
| Prior anti-CD38 exposure: yes vs no | MajesTEC-3 | 0.19 (0.05-0.67) | 0.17 (0.12-0.23) | 1.12 (0.29-4.26) |
| Prior anti-CD38 exposure: yes vs no | MonumenTAL-3, Tal-DP | 0.33 (0.14-0.76) | 0.28 (0.20-0.40) | 1.18 (0.47-2.94) |
| Prior anti-CD38 exposure: yes vs no | MonumenTAL-3, Tal-D | 0.42 (0.19-0.91) | 0.33 (0.24-0.47) | 1.27 (0.54-2.98) |
| Control regimen: PVd or DVd vs DPd | CARTITUDE-4 | 0.25 (0.11-0.53) | 0.29 (0.21-0.41) | 0.86 (0.37-2.03) |
| Control regimen: PVd or DVd vs DPd | MajesTEC-3 | 0.05 (0.01-0.24) | 0.18 (0.13-0.26) | 0.28 (0.05-1.41) |

Percentages refer to the intention-to-treat population across all arms unless stated otherwise. High-risk cytogenetics: del(17p), t(4;14), or t(14;16). In CARTITUDE-4, soft-tissue plasmacytomas include bone-based plasmacytomas with a measurable soft-tissue component, and triple-class refractoriness was reported directly; in the other trials anti-CD38-refractory disease was an exclusion criterion, so triple-class refractoriness was absent by design. APOLLO randomized DPd against Pd and contributes only the control-arm benchmark; baseline values refer to the APOLLO intention-to-treat population, whereas the benchmark median refers to its lenalidomide-refractory DPd subgroup. Sources: Einsele et al., Lancet Oncol 2026 and appendix; Costa et al., N Engl J Med 2026 and appendix; Mina et al., N Engl J Med 2026 and appendix; Dimopoulos et al., Lancet Haematol 2023.

CAR, chimeric antigen receptor; ECOG, Eastern Cooperative Oncology Group; ISS, International Staging System; NA, not applicable; NR, not reported; Pd, pomalidomide and dexamethasone.

**Table S4. Indirect contrasts and sensitivity analyses for progression-free and overall survival.**

**Primary pairwise contrasts**

| **Endpoint** | **Comparison** | **FE HR (95% CrI)** | **RE HR (95% CrI)** | **P(HR<1)** | **P(HR 0.80 to 1.25)** |
| --- | --- | --- | --- | --- | --- |
| PFS | Tec-D vs Cilta-cel | 0.62 (0.38-1.00) | 0.62 (0.21-1.81) | 0.87 | 0.19 |
| PFS | Cilta-cel vs Tal-DP | 1.04 (0.64-1.68) | 1.04 (0.36-3.02) | 0.46 | 0.43 |
| PFS | Cilta-cel vs Tal-D | 0.88 (0.55-1.40) | 0.88 (0.30-2.55) | 0.63 | 0.41 |
| PFS | Tec-D vs Tal-DP | 0.64 (0.39-1.05) | 0.64 (0.22-1.88) | 0.85 | 0.22 |
| PFS | Tec-D vs Tal-D | 0.55 (0.34-0.88) | 0.55 (0.19-1.59) | 0.91 | 0.13 |
| PFS | Tal-DP vs Tal-D | 0.85 (0.56-1.28) | 0.85 (0.56-1.28) | 0.78 | 0.58 |
| OS | Tec-D vs Cilta-cel | 0.94 (0.55-1.62) | 0.94 (0.32-2.80) | 0.56 | 0.40 |
| OS | Cilta-cel vs Tal-DP | 1.11 (0.61-2.00) | 1.11 (0.36-3.37) | 0.41 | 0.37 |
| OS | Cilta-cel vs Tal-D | 1.02 (0.57-1.83) | 1.02 (0.34-3.09) | 0.48 | 0.39 |
| OS | Tec-D vs Tal-DP | 1.04 (0.59-1.86) | 1.04 (0.34-3.15) | 0.46 | 0.39 |
| OS | Tec-D vs Tal-D | 0.96 (0.55-1.69) | 0.96 (0.32-2.89) | 0.54 | 0.39 |
| OS | Tal-DP vs Tal-D | 0.92 (0.56-1.52) | 0.92 (0.56-1.52) | 0.62 | 0.59 |

Each comparison reads agent 1 vs agent 2. P(HR<1) is the random-effects posterior probability that agent 1 has the lower hazard; P(HR 0.80 to 1.25) is the random-effects posterior probability that the hazard ratio lies between 0.80 and 1.25. Progression-free survival inputs for CARTITUDE-4 and MajesTEC-3 are the published estimates of the DPd-treated control strata. Tal-DP vs Tal-D is a within-trial comparison in MonumenTAL-3, estimated with the shared-control covariance, approximated by the inverse of the number of events in the DPd arm (136 for progression-free survival, 59 for overall survival), and without a between-trial heterogeneity term; fixed-effect and random-effects estimates therefore coincide for this comparison.

CrI, credible interval; DPd, daratumumab, pomalidomide, and dexamethasone; FE, fixed effect; HR, hazard ratio; RE, random effects; Tal-D, talquetamab plus daratumumab; Tal-DP, talquetamab plus daratumumab and pomalidomide; Tec-D, teclistamab plus daratumumab.

**Sensitivity of random-effects contrasts to the heterogeneity prior**

| **Endpoint** | **Comparison** | **Scale 0.16** | **Scale 0.32 (primary)** | **Scale 0.50** | **Scale 1.00** |
| --- | --- | --- | --- | --- | --- |
| PFS | Tec-D vs Cilta-cel | 0.62 (0.32-1.21); 0.93 | 0.62 (0.21-1.81); 0.87 | 0.62 (0.13-3.06); 0.81 | 0.62 (0.03-13.94); 0.72 |
| PFS | Cilta-cel vs Tal-DP | 1.04 (0.53-2.01); 0.45 | 1.04 (0.36-3.02); 0.46 | 1.04 (0.21-5.10); 0.47 | 1.04 (0.05-23.26); 0.48 |
| PFS | Cilta-cel vs Tal-D | 0.88 (0.46-1.69); 0.67 | 0.88 (0.30-2.55); 0.63 | 0.88 (0.18-4.32); 0.61 | 0.88 (0.04-19.70); 0.57 |
| PFS | Tec-D vs Tal-DP | 0.64 (0.33-1.26); 0.92 | 0.64 (0.22-1.88); 0.85 | 0.64 (0.13-3.17); 0.80 | 0.64 (0.03-14.45); 0.71 |
| PFS | Tec-D vs Tal-D | 0.55 (0.28-1.05); 0.97 | 0.55 (0.19-1.59); 0.91 | 0.55 (0.11-2.68); 0.86 | 0.55 (0.02-12.24); 0.76 |
| PFS | Tal-DP vs Tal-D | 0.85 (0.56-1.28); 0.78 | 0.85 (0.56-1.28); 0.78 | 0.85 (0.56-1.28); 0.78 | 0.85 (0.56-1.28); 0.78 |
| OS | Tec-D vs Cilta-cel | 0.94 (0.47-1.91); 0.57 | 0.94 (0.32-2.80); 0.56 | 0.94 (0.19-4.71); 0.55 | 0.94 (0.04-21.30); 0.53 |
| OS | Cilta-cel vs Tal-DP | 1.11 (0.52-2.34); 0.39 | 1.11 (0.36-3.37); 0.41 | 1.11 (0.22-5.60); 0.42 | 1.11 (0.05-25.18); 0.45 |
| OS | Cilta-cel vs Tal-D | 1.02 (0.49-2.14); 0.48 | 1.02 (0.34-3.09); 0.48 | 1.02 (0.20-5.15); 0.49 | 1.02 (0.04-23.18); 0.49 |
| OS | Tec-D vs Tal-DP | 1.04 (0.50-2.17); 0.45 | 1.04 (0.34-3.15); 0.46 | 1.04 (0.21-5.26); 0.47 | 1.04 (0.05-23.68); 0.48 |
| OS | Tec-D vs Tal-D | 0.96 (0.46-1.99); 0.55 | 0.96 (0.32-2.89); 0.54 | 0.96 (0.19-4.83); 0.53 | 0.96 (0.04-21.79); 0.52 |
| OS | Tal-DP vs Tal-D | 0.92 (0.56-1.52); 0.62 | 0.92 (0.56-1.52); 0.62 | 0.92 (0.56-1.52); 0.62 | 0.92 (0.56-1.52); 0.62 |

Cells give the random-effects HR (95% CrI) followed by the posterior probability that agent 1 has the lower hazard. The scale is the half-normal prior on tau (log-hazard-ratio scale). Overall survival contrasts, not shown, excluded 1 under no prior. The within-trial Tal-DP vs Tal-D comparison carries no heterogeneity term and is invariant to the prior.

**Tipping-point analysis**

| **Endpoint** | **Comparison** | **FE HR (95% CrI)** | **Smallest tau at which the 95% interval includes 1** | **Prior probability of a smaller tau (primary prior)** |
| --- | --- | --- | --- | --- |
| PFS | Tec-D vs Tal-D | 0.55 (0.34-0.88) | 0.14 | 0.33 |

Only the single fixed-effect contrast whose 95% interval excluded 1 is listed. Tau was fixed across a grid from 0 to 0.50; the prior probability of a smaller tau refers to the primary half-normal prior, scale 0.32.

**Intention-to-treat sensitivity analysis**

| **Endpoint** | **Comparison** | **Primary FE HR (95% CrI)** | **ITT FE HR (95% CrI)** | **ITT RE HR (95% CrI)** | **P(HR<1)** |
| --- | --- | --- | --- | --- | --- |
| PFS | Tec-D vs Cilta-cel | 0.62 (0.38-1.00) | 0.59 (0.38-0.90) | 0.59 (0.20-1.68) | 0.90 |
| PFS | Cilta-cel vs Tal-DP | 1.04 (0.64-1.68) | 1.04 (0.66-1.62) | 1.04 (0.36-2.99) | 0.46 |
| PFS | Cilta-cel vs Tal-D | 0.88 (0.55-1.40) | 0.88 (0.57-1.36) | 0.88 (0.31-2.52) | 0.64 |
| PFS | Tec-D vs Tal-DP | 0.64 (0.39-1.05) | 0.61 (0.38-0.98) | 0.61 (0.21-1.77) | 0.88 |
| PFS | Tec-D vs Tal-D | 0.55 (0.34-0.88) | 0.52 (0.33-0.82) | 0.52 (0.18-1.49) | 0.93 |
| PFS | Tal-DP vs Tal-D | 0.85 (0.56-1.28) | 0.85 (0.56-1.28) | 0.85 (0.56-1.28) | 0.78 |
| OS | Tec-D vs Cilta-cel | 0.94 (0.55-1.62) | 0.84 (0.51-1.38) | 0.84 (0.29-2.45) | 0.67 |
| OS | Cilta-cel vs Tal-DP | 1.11 (0.61-2.00) | 1.17 (0.66-2.06) | 1.17 (0.39-3.52) | 0.36 |
| OS | Cilta-cel vs Tal-D | 1.02 (0.57-1.83) | 1.08 (0.62-1.88) | 1.08 (0.36-3.23) | 0.43 |
| OS | Tec-D vs Tal-DP | 1.04 (0.59-1.86) | 0.98 (0.55-1.73) | 0.98 (0.32-2.95) | 0.52 |
| OS | Tec-D vs Tal-D | 0.96 (0.55-1.69) | 0.90 (0.52-1.57) | 0.90 (0.30-2.70) | 0.60 |
| OS | Tal-DP vs Tal-D | 0.92 (0.56-1.52) | 0.92 (0.56-1.52) | 0.92 (0.56-1.52) | 0.62 |

The primary analysis uses the DPd-stratum estimates for CARTITUDE-4 and MajesTEC-3. The intention-to-treat analysis uses the published estimates including PVd- and DVd-treated controls: CARTITUDE-4 PFS 0.29, OS 0.55; MajesTEC-3 PFS 0.17, OS 0.46. MonumenTAL-3 used DPd as the only control and is unchanged. ITT, intention to treat.

**Table S5. Anti-CD38-naïve harmonization (PFS): intention-to-treat versus harmonized estimates.** Only the cilta-cel edges change; P(HR<1) is the random-effects posterior probability that agent 1 has the lower hazard.

| **Comparison** | **ITT FE HR (95% CrI)** | **Harmonized FE HR (95% CrI)** | **Harmonized RE HR (95% CrI)** | **P(HR<1)** |
| --- | --- | --- | --- | --- |
| Tec-D vs Cilta-cel | 0.62 (0.38-1.00) | 0.58 (0.36-0.95) | 0.58 (0.20-1.70) | 0.89 |
| Cilta-cel vs Tal-DP | 1.04 (0.64-1.68) | 1.11 (0.68-1.81) | 1.11 (0.38-3.24) | 0.40 |
| Cilta-cel vs Tal-D | 0.88 (0.55-1.40) | 0.94 (0.58-1.52) | 0.94 (0.32-2.74) | 0.56 |
| Tec-D vs Tal-DP | 0.64 (0.39-1.05) | 0.64 (0.39-1.05) | 0.64 (0.22-1.88) | 0.85 |
| Tec-D vs Tal-D | 0.55 (0.34-0.88) | 0.55 (0.34-0.88) | 0.55 (0.19-1.59) | 0.91 |
| Tal-DP vs Tal-D | 0.85 (0.53-1.36) | 0.85 (0.52-1.37) | 0.85 (0.29-2.47) | 0.66 |

**Table S6. Adverse events in the T-cell-redirecting arms of the included randomized trials (descriptive summary).**

| **Adverse event** | **CARTITUDE-4 (cilta-cel)** | **MajesTEC-3 (Tec-D)** | **MonumenTAL-3 (Tal-DP)** | **MonumenTAL-3 (Tal-D)** |
| --- | --- | --- | --- | --- |
| No. of patients, safety (infused) | 208 (176) | 283 | 276 | 274 |
| Median follow-up, months | 33.6 | 34.5 | 24.6 | 24.6 |
| Median treatment duration, months | single infusion | 32.4 | 21.9 | 21.5 |
| **Overall, %** | | | | |
| Grade 3 or 4 adverse event | 89 | 95 | 97 | 79 |
| Serious adverse event | 47 | 71 | 63 | 53 |
| Fatal (grade 5) adverse event | 7 | 7 | 2 | 4 |
| **Hematologic, % any grade (grade 3 or higher)** | | | | |
| Neutropenia | 90 (90) | 78 (76) | 84 (76) | 41 (29) |
| Anemia | 54 (36) | 39 (21) | 49 (24) | 39 (15) |
| Thrombocytopenia | 54 (42) | 36 (19) | 49 (24) | 34 (13) |
| Lymphopenia | 22 (21) | 22 (21) | 36 (31) | 30 (25) |
| **Infections, % any grade (grade 3 or higher)** | | | | |
| Any infection | 63 (28) | 97 (54) | 87 (38) | 84 (29) |
| Pneumonia | 7 (4) | 23 (17) | NR | NR |
| **Immune-effector events, % any grade (grade 3 or higher)** | | | | |
| Cytokine release syndrome (cilta-cel: infused) | 75 (1) | 60 (0) | 68 (0.7) | 58 (0.7) |
| ICANS (cilta-cel: infused) | 5 (0) | 1 (0.4) | 3 (1.1) | 2 (0) |
| **Agent-associated events, % any grade (grade 3 or higher)** | | | | |
| Dysgeusia or taste change | NR | NR | 73 | 75 |
| Nonrash skin event | NR | NR | 69 (2) | 65 (3) |
| Nail-related event | NR | NR | 56 (0.4) | 57 (0.4) |
| Weight decreased | NR | NR | 46 (8) | 38 (5) |
| Parkinsonism (cilta-cel: infused) | 1 (0) | 0 | 0 | 0 |
| Cranial-nerve palsy (cilta-cel: infused) | 9 | 0 | 0 | 0 |
| Ataxia and balance disorders | NR | NR | 15 (3) | 12 (2) |
| **Hypogammaglobulinemia, % (reported under two non-comparable definitions)** | | | | |
| IgG below 400 mg/dL or investigator-reported | NR | 84.5 | 81.5 | 70.1 |
| Adverse-event term, any grade (grade 3 or higher) | 46 (8) | 69 (6) | 49 (3) | 42 (3) |

Values are percent of the safety population (any grade, with grade 3 or higher in parentheses where reported); rates were not compared statistically because follow-up, treatment exposure, safety populations, and ascertainment differ across trials. NR, not reported.

For cilta-cel, most adverse events are reported in the safety population (n=208); the events marked “cilta-cel: infused” (cytokine release syndrome, ICANS, parkinsonism, cranial-nerve palsy) are reported in the infused population (n=176).

Hypogammaglobulinemia is shown in two separate rows because the trials used different definitions: an IgG threshold of less than 400 mg/dL or investigator report, and an adverse-event term. The two rows are based on different definitions and are not comparable between rows.

**Table S7. Cost inputs (US wholesale acquisition cost, 80-kg patient) for the cost-consequence model.**

| **Item** | **Value** | **Basis** |
| --- | --- | --- |
| Daratumumab | $10,985 | per dose |
| Teclistamab | $69.30 | per mg |
| Talquetamab | $293.00 | per mg |
| Pomalidomide | $25,000 | per cycle |
| Ciltacabtagene autoleucel (product) | $465,000 | one-time |
| Cilta-cel loaded total | $547,940 | product + apheresis + lymphodepletion + bridging + administration |
| Cycle length | 28 days |  |

**Supplementary Figures**

**Figure S1. Risk of bias for progression-free survival (Cochrane RoB 2).**

Open-label design yields an overall rating of some concerns, driven by the deviations-from-intended-intervention and outcome-measurement domains.

**Figure S2. Kaplan-Meier reconstruction validation, CARTITUDE-4.**

Reconstructed pseudo-patient progression-free-survival curves overlaid on the published curves; the exact variant reproduced the reported event totals (89 cilta-cel; 153 standard-of-care).

**Figure S3. Frequentist network results.**

Frequentist confirmatory network meta-analysis versus DPd, for progression-free survival and overall survival. Squares are hazard ratios versus the shared DPd-type control with 95% confidence intervals, colored by agent; P-score (the mean probability of outranking a competitor) is given at right. Because the network is a star with one trial per edge, between-trial heterogeneity is not estimable and the random-effects model reduces to the fixed-effect model, so these estimates coincide with the Bayesian fixed-effect contrasts. Tec-D, teclistamab-daratumumab; Tal-DP, talquetamab-daratumumab-pomalidomide; Tal-D, talquetamab-daratumumab.

**Figure S4. Frequentist network meta-analysis league table for progression-free and overall survival.**

Pairwise indirect hazard ratios from the frequentist (graph-theoretical) network meta-analysis, for progression-free survival (A) and overall survival (B). Each cell gives the hazard ratio and 95% confidence interval for the column agent versus the row agent; a hazard ratio below 1 favors the column agent. Cells are shaded by effect size (green toward the column agent, orange toward the row agent), and bold marks intervals excluding 1. Because the network is a star with a single trial per edge, no within-network heterogeneity is estimable and the random-effects model reduces to the fixed-effect model, so these estimates coincide with the Bayesian fixed-effect contrasts.

**Figure S5. Between-trial heterogeneity.**

**Figure S6. Sensitivity analysis restricted to subgroups without prior anti-CD38 exposure.**

**Figure S7. Overall-survival difference in restricted mean survival time (ΔRMST) versus the restriction horizon, by agent.**

The cilta-cel advantage accrues later, increasing with the horizon; all pairwise indirect RMST contrasts include zero across horizons.

**Figure S8. Second primary malignancies.**

**Figure S9. Cost per progression-free month under standardized and trial-specific denominators.**
